# Effect of Fruit Powders as Natural Alternatives to Sodium Nitrite on Lipid Oxidation in Clean-Label Salami

**DOI:** 10.3390/foods14132262

**Published:** 2025-06-26

**Authors:** Adriana-Ioana Moraru Manea, Ileana Cocan, Delia-Gabriela Dumbrava, Mariana-Atena Poiana

**Affiliations:** Faculty of Food Engineering, University of Life Sciences “King Mihai I” from Timisoara, Calea Aradului No. 119, 300645 Timisoara, Romania; adriana.manea@usvt.ro (A.-I.M.M.); ileanacocan@usvt.ro (I.C.); deliadumbrava@usvt.ro (D.-G.D.)

**Keywords:** fruit powders, natural antioxidants, clean-label salami, nitrite substitutes, lipid oxidation

## Abstract

Public concerns about the health risks of synthetic antioxidants have prompted the meat industry to look for natural alternatives rich in phenols with strong antioxidant properties. This study investigates the use of blackcurrant (BCP), lingonberry (LP), and sour cherry (SCP) powders as natural substitutes for synthetic nitrites in reformulating two clean-label salami types, smoked and cooked and smoked and scalded, with a focus on their effects on oxidative stability during processing and refrigerated storage (4 °C). Nitrite-free formulations were prepared with each fruit powder at three inclusion levels to provide total phenolic contents of 90, 200, and 300 mg gallic acid equivalents (GAE)/kg of processed meat. A nitrite-containing control (90 mg/kg) and an additive-free control were included for comparison. The phytochemical profiles of powders were characterized by total phenolic, flavonoid, monomeric anthocyanin contents, and L-ascorbic acid levels. Antioxidant activity was assessed via 1,1-diphenyl-2-picrylhydrazyl (DPPH) radical scavenging and ferric reducing antioxidant power (FRAP) assays. Salami samples were analyzed for proximate composition, and lipid oxidation was monitored at 0, 15, and 30 days of storage using peroxide value, inhibition of oxidation, p-anisidine value, TOTOX, and thiobarbituric acid value. Fruit powders demonstrated dose- and type-dependent inhibition of primary and secondary lipid oxidation, enhancing oxidative stability during processing and storage. After 30 days of storage, oxidation markers in fruit-enriched salami remained below recommended thresholds, confirming effective control of lipid oxidation. The inhibitory potential followed the order BCP > LP > SCP, consistent with antioxidant profiles as reflected by DPPH and FRAP values. BCP at 300 mg GAE/kg showed a stronger lipid oxidation inhibition than sodium nitrite. Promising improvements in lipid oxidation resistance were also observed with LP at 300 mg GAE/kg and BCP at 200 mg GAE/kg. These findings highlight the potential of fruit-derived antioxidants to support the development of more sustainable, value-added meat products without compromising quality.

## 1. Introduction

Meat products are vital for human nutrition, providing excellent sources of proteins, fats, essential amino acids, vitamins, minerals, and other critical nutrients. However, their complex composition makes them susceptible to significant chemical transformations during processing and storage, potentially forming harmful substances like nitrosamines and lipid oxidation products [1]. Oxidation of both lipids and proteins not only diminishes shelf life but can also generate detrimental end-products [2]. Specifically, lipid oxidation is a primary driver of quality degradation in meat products during storage and processing. This complex process initiates with the peroxidation of unsaturated fatty acids in phospholipid membranes, yielding hydroperoxides as primary oxidation products. Subsequently, these compounds decompose into secondary compounds such as aldehydes, ketones, alkenes, and alcohols, negatively impacting both the sensory qualities and nutritional value of meat and meat products [3,4]. Currently, synthetic preservatives are widely employed to protect foods from quality deterioration. They mitigate the damaging effects of free radicals in meat systems, thereby minimizing oxidative degradation during processing and storage and consequently extending product shelf life [5,6].

Synthetic nitrates and nitrites (as potassium or sodium salts) are widely used in processed meat for their vital role in quality, particularly color stability, and their ability to retard bacterial spoilage and lipid oxidation [7]. In cured meats, nitrites exert a protective antioxidative effect as nitric oxide (NO) binds to heme iron, preventing lipid peroxidation and enhancing storage stability. Despite these benefits, nitrite remains one of the most controversial additives due to the toxicity associated with the formation of nitrosamines through nitrosation reactions between secondary amines and nitrosating agents such as nitrates, nitrites, or nitrogen oxides during meat processing. This issue highlights the need to shift towards clean-label strategies that address current sustainability and food safety concerns [8,9]. Consumer health concerns have become increasingly prominent in recent years, reflected in a willingness to pay more for nitrite-free meat products [10,11]. This trend has led to the reformulation of meat products, specifically by reducing or substituting nitrites through the incorporation of plant materials rich in phytochemicals. To address this challenge, natural extracts and ingredients offer a valuable alternative to conventional synthetic additives, with the potential to effectively slow product quality deterioration [12,13,14,15]. Common phenolic compounds are key plant components, exhibiting strong antioxidant capabilities by scavenging reactive nitrogen/oxygen species and free radicals, inhibiting free radical-forming enzymes, binding metals, and activating antioxidant enzymes. In meat products, polyphenols effectively inhibit oxidation, preventing discoloration and quality deterioration [16]. Numerous studies attest to the efficacy of natural antioxidants in meat products [17,18]. Replacing synthetic nitrates and antioxidants with plant-based alternatives (powders, extracts, and other natural derivatives) has been shown to strongly inhibit protein and lipid oxidation, slow degradation processes, retard off-flavor development (rancidity), improve color stability, enhance microbiological quality, and extend the shelf life of fresh and processed meat products. Crucially, these natural alternatives achieve comparable effectiveness to synthetic preservatives without compromising sensory or nutritional properties [19]. Incorporating plant-derived bioactive compounds with antioxidant function into meat products can preserve their composition and quality while offering health benefits to consumers [20]. Fruit powders, in particular, with their significant content of high-value phenolic compounds and other bioactive ingredients, can effectively prevent the initiation or propagation of lipid oxidation reactions. Multiple research efforts confirm the efficacy of these plant-derived compounds. For instance, mulberry fruit powder significantly extended the shelf life of minced beef due to its antioxidant properties [21]. Similarly, fruit powders derived from red grapes, gooseberry, and tomato have been shown to preserve the quality of restructured chicken slices for up to 20 days under refrigerated storage conditions [22]. Plant-derived powders, rich in complex phytochemical compounds, offer a viable alternative to individual synthetic antioxidants for extending the shelf life of meat products and may also have associated nutritional and health benefits. Certain vegetable powders have been shown to significantly enhance the oxidative stability of turkey meat patties by 20–30% under accelerated oxidation conditions [23]. It has also been reported that fortifying beef burgers with blueberries resulted in improved sensory quality and increased primary and secondary lipid oxidation stability, demonstrating its high potential as a natural functional ingredient with preservative capacity [24]. In general, powdered plant materials have notable antimicrobial and antioxidant properties in minced meat products [25]. The results of the study conducted by Martínez-Zamora et al. [26] revealed the high potential of spices, fruits, and vegetables to limit the oxidative deterioration of lipids and proteins, making them a suitable alternative to synthetic counterparts.

Our previous research highlighted the potential of tomato processing byproducts [27] and bell pepper processing byproducts [28] as promising substitutes for sodium nitrite. These byproducts can ensure lipid oxidative stability during the cold storage of sausages for 20 days, contributing to the development of value-added meat products. The application of natural extracts and spices, fruits, and vegetables in formulating clean-label meat products as substitutes for synthetic nitrites/nitrates and antioxidants is a growing trend. However, limited studies document their effect on the oxidative stability of meat products during prolonged cold storage. Addressing the challenges previously outlined, this study aimed to evaluate the efficacy of blackcurrant, lingonberry, and sour cherry powders as natural alternatives to nitrites in the reformulation of two distinct salami types intended to be clean-label, smoked and cooked, and smoked and scalded, focusing on their effects on oxidative stability during processing and refrigerated storage (4 °C). Our central hypothesis is that the bioactive compounds inherent in these fruit powders could serve as a viable replacement for synthetic additives commonly used in meat products, thereby not only addressing potential health concerns but also significantly improving the functional profile, sustainability, and overall quality of the final product. Specifically, this research focused on: (1) determining the phytochemical content and antioxidant activities of the fruit powders, and (2) evaluating their ability to effectively inhibit lipid oxidation throughout both the processing and cold storage (up to 30 days) of the salami samples. Nitrite-free salami formulations were developed by incorporating fruit powders at doses calculated to provide total phenolic contents of 90, 200, and 300 mg gallic acid equivalents (GAE)/kg of processed meat. The salami samples were analyzed for their proximate composition, and the progression of lipid oxidation was systematically evaluated using specific chemical indices at 0, 15, and 30 days of storage at 4 °C.

## 2. Materials and Methods

### 2.1. Materials

Fresh meat and pork fat for the salami recipe were sourced from Comtim Romania SRL (Timis, Romania). Other ingredients were procured from specific suppliers: salt from Salrom-SNS SA (Bucharest, Romania), nitrite salt from Daz Activ Trade SRL (Botoșani, Bucharest, Romania), and spices from Profood Rom SRL (Miercurea Ciuc, Harghita County, Romania). The spice blend included granulated garlic, ground thyme, ground white and black pepper, ground allspice, sweet paprika, hot paprika, ground nutmeg, ground coriander, and ground cumin.

Frozen fruits were procured from Romanian producers. Blackcurrants (*Ribes nigrum* L.) came from SC Forelit (Salard, Bihor County, Romania), lingonberries (*Vaccinium vitis-idaea* L.) from SC Vladalex Impex (Targu Mures, Mures County, Romania), and sour cherries (*Prunus cerasus* L.) from SC Gradina Padurii (Oradea, Bihor County, Romania).

For chemical analyses, analytical grade reagents were supplied by Chemical Company (Iasi, Romania), Sigma-Aldrich (St. Louis, MO, USA), Chimreactiv (Bucharest, Romania), and Adra Chim (Bucharest, Romania).

### 2.2. Obtaining the Fruit Powder

Prior to processing, the frozen fruit samples were allowed to thaw at room temperature for approximately two hours. Subsequently, the samples were subjected to convective drying in a BINDER drying chamber (Binder GmbH, Tuttlingen, Germany) at 60 °C for a total of 15 h, divided into three 5 h sessions conducted over three consecutive days. Drying at this temperature is known to limit enzymatic degradation, thereby improving the preservation of polyphenolic compounds and maintaining the functional quality of the dried plant matrix [29]. The dried fruits reached a final moisture content below 5%, with final values of 4.59% for sour cherry, 4.93% for blackcurrant, and 4.71% for lingonberry. Achieving such low moisture levels is essential for microbial stability, as water activity values in the range of 0.2–0.3, usually associated with a moisture content below 5%, are considered microbiologically safe [30]. After drying, the samples were cooled to 20 °C and ground to a fine powder using a Grindomix GM 2000 laboratory mill (Retsch GmbH, Germany), then passed through a 60-mesh sieve to ensure uniform particle size. The powders obtained from blackcurrants (BCP), lingonberry (LP), and sour cherries (SCP) were vacuum-packed in polypropylene bags and stored at room temperature in the dark until further analysis or application.

### 2.3. Manufacture of Salami Formulas

This study focused on developing two salami types formulated as clean-label products by replacing sodium nitrite with sour cherry (SCP), blackcurrant (BCP), or lingonberry (LP) powders. The two types, smoked and cooked (SI) and smoked and scalded (SII), differ in their specific recipes and processing parameters. Both product types undergo the same overall processing time, but SI is subjected to a final heat treatment at 72 °C with 5% relative air humidity, while SII is treated at the same temperature under approximately 80% relative humidity, which allows a comparative evaluation of the antioxidative efficacy of fruit powders in mitigating oxidative degradation under typical industrial meat production conditions. All salami formulations were produced under standard conditions at the S.C. Cavarantana meat processing unit in the village of Cavaran, Caraș-Severin County, Romania, following the traditional manufacturing procedures employed at this facility. For each salami type (SI and SII), 11 distinct experimental variants were prepared. The design included a positive control containing 90 mg sodium nitrite per kilogram of processed meat and a negative control without nitrite. The positive control samples used a salt mixture containing 0.5% (g/g) sodium nitrite, while the negative control samples used salt without added sodium nitrite. In the remaining variants, sodium nitrite was entirely replaced with fruit powders incorporated at concentrations calculated to provide target total phenolic contents (TPC) of 90, 200, or 300 mg gallic acid equivalents (GAE) per kilogram of processed meat. The specific amount of each fruit powder (SCP, BCP, and LP) incorporated into the recipes was determined by its individual total phenolic compound content. The minimum total phenolic content (TPC) level of 90 mg GAE/kg in the processed meat, supplied by the fruit powders, was selected to correspond to the sodium nitrite concentration used in the traditional SI and SII recipes (90 mg/kg). The selection of these fruit powder doses for the sausage formulation was based on our previous studies, where supplementation with tomato byproducts [27] and yellow and red bell pepper [28], providing total phenolic content levels of 90, 180, and 270 mg GAE/kg of processed meat, yielded promising results in limiting oxidative degradation during processing and 21 days of cold storage. All salami variants were prepared in a cold room maintained at a temperature below 10 °C. The codes for the salami formulations are defined as follows:

SI-C: Nitrite-free smoked and cooked salami (negative control sample);
SI-CN: Smoked and cooked salami with added sodium nitrite (positive control);
SI-SCP90, SI-SCP200, SI-SCP300: Smoked and cooked nitrite-free salami with sour cherry powder added to provide a TPC of 90, 200, and 300 mg GAE/kg of processed meat;
SI-BCP90, SI-BCP200, SI-BCP300: Smoked and cooked nitrite-free salami with blackcurrant powder added to provide a TPC of 90, 200, and 300 mg GAE/kg of processed meat;
SI-LP90, SI-LP200, SI-LP300: Smoked and cooked nitrite-free salami with lingonberry powder added to provide a TPC of 90, 200, and 300 mg GAE/kg of processed meat;
SII-C: Nitrite-free smoked and scalded salami (negative control sample);
SII-CN: Smoked and scalded salami with added sodium nitrite (positive control);
SII-SCP90, SII-SCP200, SII-SCP300: Smoked and scalded nitrite-free salami with sour cherry powder added to provide a TPC of 90, 200, and 300 mg GAE/kg of processed meat;
SII-BCP90, SII-BCP200, SII-BCP300: Smoked and scalded nitrite-free salami with blackcurrant powder added to provide a TPC of 90, 200, and 300 mg GAE/kg of processed meat;
SII-LP90, SII-LP200, SII-LP300: Smoked and scalded nitrite-free salami with lingonberry powder added to provide a TPC of 90, 200, and 300 mg GAE/kg of processed meat.

Ingredient quantities for SI salami (per kg of pork/fat mixture) are presented in Table 1.

The spice mixture used in the SI formulation contained 7 g of garlic granules, 3 g of ground thyme, 3 g of ground white pepper, and 2 g of ground allspice.

Table 2 details the ingredients per kg of pork/fat mixture in SII salami.

The spice mixture used in SII formulations contained 9 g of garlic granules, 10 g of sweet paprika, 1 g of hot paprika, 3 g of ground black pepper, 2 g of ground nutmeg, 1 g of ground coriander, and 2 g of ground cumin.

The technological flow followed for SI production involves cutting pork meat and fat into pieces of approximately 50 g, mincing them using a meat grinder with a 5 mm sieve (Luohe, YGM-100, Henan, China), adding the other ingredients in the recipe, and homogenizing with a mixer (Inotec IM-150E, Reutlingen, Germany) for 10 min. The resulting mixture is stuffed into collagen membranes with a diameter of 45 mm using a filling machine (Albert Handtmann, VF-608, 88400 Biberach an der Riss, Germany), and the resulting salami sticks are sealed at both ends with metal clips using a clip sealing machine (Tipper Tie Technopack, KDCVT 400, 21509 Glinde, Germany). The salamis are left to mature in a cold room for 24 h at a temperature of 4 °C. The heat treatment of the raw salami formulations was carried out in a smoke oven (Doleschal, SC6001, A-4400 Steyr, Austria), undergoing the following phases: preheating at 60 °C for 15 min, drying at 70 °C for 30 min, smoking at 70 °C for 30 min, and cooking at 72 °C and 5% relative air humidity until an internal temperature of 70 °C is reached in the salami.

SII followed the same manufacturing process as SI, except for the final heat treatment stage, which was conducted at 72 °C in an environment with approximately 80% relative air humidity until the internal temperature of the salami reached 70 °C. After heat treatment, all salami formulas are cooled, packaged in oxygen-permeable bags, and stored in a cold room at 4 °C and a relative air humidity of 70–80%, after being divided according to each experimental time (0, 15, and 30 days). All formulations were replicated independently twice. For each replicate, six pieces of salami were produced per treatment. The salami has a recommended shelf life of 30 days.

**Figure 1:**
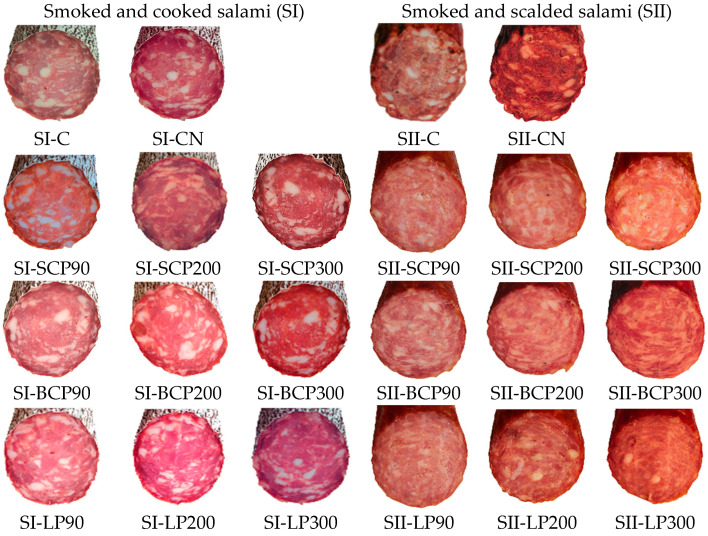
Salami formulas after 30 days of cold storage. SI-C, SII-C: Nitrite-free SI and SII salami; SI-CN, SII-CN: SI and SII salami with added sodium nitrite; SI-SCP90, SI-SCP200, SI-SCP300, SII-SCP90, SII-SCP200, SII-SCP300: Nitrite-free SI and SII salami with sour cherry powder added to provide a TPC of 90, 200 and 300 mg GAE/kg of meat; SI-BCP90, SI-BCP200, SI-BCP300, SII-BCP90, SII-BCP200, SII-BCP300: Nitrite-free SI and SII salami with blackcurrant powder added to provide a TPC of 90, 200 and 300 mg GAE/kg of meat; SI-LP90, SI-LP200, SI-LP300, SII-LP90, SII-LP200, SII-LP300: Nitrite-free SI and SII salami with lingonberry powder added to provide a TPC of 90, 200 and 300 mg GAE/kg of meat.

Figure 1 displays the salami samples after this 30-day cold storage period.

The salami samples from day 0 were tested for proximate chemical composition and lipid oxidation, while samples taken after 15 and 30 days of storage were tested to assess the progression of lipid oxidation based on specific chemical indices, such as the peroxide value (PV), para-anisidine value (pAV), and thiobarbituric acid value (TBA). The total oxidation value (TOTOX) and oxidation inhibition (IO, %) were also calculated.

### 2.4. Phytochemical Profile and Antioxidant Activity of Fruit Powder

#### 2.4.1. Obtaining the Extract for Evaluating the Total Phenolic Content, Total Flavonoid Content, and Antioxidant Activity

Alcoholic extracts were prepared by adding 10 mL of 70% (*v*/*v*) ethanol to 0.5 g of fruit powders (SCP, BCP, and LP), following the method of Litwinek et al. [31] with minor modifications. Extraction was carried out for 2 h at ambient temperature under continuous stirring with a magnetic stirrer (IDL GmbH & Co KG, Nidderau, Germany), after which the mixtures were centrifuged for 10 min at 10,000 rpm (Hettich EBA 21, Andreas Hettich GmbH & Co. KG., Tuttlingen, Germany). The supernatant was collected, and the remaining residue was re-extracted with a new portion of 70% ethanol (*v*/*v*) for another 60 min under the same conditions. The collected supernatants were combined, and the volume of the mixture was adjusted to 20 mL with 70% ethanol (*v*/*v*) and stored at −20 °C in the dark until analysis of total phenolic content, total flavonoid content, and antioxidant activity. The extraction procedure was carried out in triplicate for each fruit powder.

#### 2.4.2. Evaluation of Total Phenolic Content

The total phenolic content (TPC) was quantified according to the Folin–Ciocalteau method [32]. Before analysis, the alcoholic extracts from the fruit powder were diluted in a ratio of 1:10 (*v*/*v*) with distilled water. Subsequently, 0.5 mL of diluted extracts was combined with 2.5 mL of Folin-Ciocalteu reagent, previously diluted in a ratio of 1:10 *v*/*v* with distilled water, and 2 mL of 7.5% Na_2_CO_3_ solution. The resulting mixture was incubated at 50 °C for 30 min, followed by measurement of the absorbance at 750 nm using the SPECORD 205 UV-Vis spectrophotometer (Analytik Jena Inc., Jena, Germany) against a control sample prepared under the same conditions. A gallic acid calibration curve was generated by plotting values of standards’ absorbances versus their concentrations in the range 0.1–1.0 µM GAE/mL. The TPC in extracts was calculated from the regression equation, and the results were converted to mg gallic acid equivalent (GAE)/g dry weight (d.w.) of fruit powder.

#### 2.4.3. Evaluation of Total Flavonoid Content (TFC)

Total flavonoid content in the fruit powder extracts was quantified using an adapted colorimetric assay [33]. In brief, a 3.0 mL portion of the ethanol extract, previously obtained, was introduced to a mixture containing 4.5 mL of deionized water and 1.0 mL of a 0.3% (*w*/*v*) aqueous solution of sodium nitrite. This initial mixture underwent a 6 min incubation period at a controlled temperature of 20 °C. Subsequently, 1.0 mL of 10% (*w*/*v*) aluminum nitrate solution was incorporated, followed by an additional incubation period of 6 min. The procedure continued with the addition of 10.0 mL of 4% (w/w) sodium hydroxide solution, after which the final volume of the reaction mixture was adjusted to 25.0 mL using a 70% ethanol solution (*v*/*v*) and allowed to stand at room temperature for 15 min to complete the reaction. The absorbance was measured at 510 nm against a 70% (*v*/*v*) ethanol blank using a UV-Vis spectrophotometer. A calibration curve was generated employing a series of quercetin (QE) standard solutions, covering a concentration range of 0.5 to 50 μg/mL. The total flavonoid content of fruit powder was ultimately expressed as milligrams of quercetin equivalents per 100 g of dry weight (mg QE/100 g d.w.).

#### 2.4.4. Evaluation of Monomeric Anthocyanin Content

The anthocyanin content in fruit powder extract was determined spectrophotometrically as monomeric anthocyanin by the pH-differential method [34]. Fruit powder extracts were prepared by mixing 0.25 g of fruit powder with 5 mL of a 0.1 N HCl and 96% (*v*/*v*) ethanol mixture (1:9, *v*/*v*). The extraction proceeded for one hour under continuous stirring. The suspension was then centrifuged for 5 min at 5000 rpm, and the residue obtained after removing the supernatant was re-extracted twice under the same extraction conditions. The combined supernatants were adjusted to a final volume of 15 mL. The extraction was performed in triplicate for each fruit powder. The alcoholic extracts obtained from SCP, BCP, and LP were diluted 1:5, *v*/*v* in 0.025 M potassium chloride buffer (pH 1.0) and 0.4 M sodium acetate buffer (pH 4.5) and the absorbance of each was measured at both 520 and 700 nm with a UV-Vis spectrophotometer Specord 205 (Analytik Jena Inc., Jena, Germany) against distilled water as a blank in a glass cuvette with an optical path length of 10 mm. TMA content was calculated according to Darniadi et al. [34] and expressed as mg cyanidin-3-glucoside equivalents (C3G) per 100 g d.w. using a molar extinction coefficient of 26 900 L/cm·mol and molecular weight of 449.2 g/mol for C3G.

#### 2.4.5. Determination of L-Ascorbic Acid

L-Ascorbic acid (AsAc) or vitamin C content was measured by titrimetric method with 2,6-dichlorophenol-indophenol sodium salt solution. AsAc was extracted by homogenizing 1 g of the fruit powder with 20 mL of 2% (*w*/*v*) oxalic acid solution. The mixture was allowed to stand for about 1 h at 20 °C and filtered through Whatman filter paper No 2 to remove any remaining plant material. The filtrate was mixed with the kaolin decolorizer and re-filtered [35]. Next, 10 mL of the obtained clear filtrate was titrated with 0.025% (*m*/*v*) 2,6-dichloroindophenol solution until a pink color was obtained. The final AsAc content was calculated according to Žlabur [36] and expressed as mg/100 g d.w.

#### 2.4.6. Evaluation of the Antioxidant Activity of Fruit Powder via 1,1-Diphenyl-2-picrylhydrazyl (DPPH) Method

The antioxidant activity of the fruit powder extracts, specifically their ability to scavenge free radicals, was quantified via the 1,1-diphenyl-2-picrylhydrazyl (DPPH) assay [37] with a 0.1 mM DPPH solution in 70% (*v*/*v*) ethanol. The previously obtained ethanolic extracts of each fruit powder were diluted with 70% (*v*/*v*) ethanol at a volumetric ratio of 1:50. Subsequently, a 1.0 mL aliquot of the diluted extracts was combined with 2.5 mL of the 0.1 mM DPPH solution in 70% (*v*/*v*) ethanol. The resulting mixtures were homogenized using a hot plate stirrer (IDL GmbH & Co KG, Nidderau, Germany) and then subjected to a 30 min incubation period in darkness at 20 °C. The absorbance of each incubated mixture was measured at a wavelength of 517 nm against 70% (*v*/*v*) ethanol as the reference blank. Under identical operational conditions, a control sample, consisting of 1.0 mL of 70% (*v*/*v*) ethanol mixed with 2.5 mL of the 0.1 mM DPPH solution in 70% (*v*/*v*) ethanol, was also prepared and analyzed. The DPPH radical scavenging activity was calculated as shown in Equation (1), where A_control_ and A_sample_ represent the absorbance values of the control and sample, respectively.

(1) $$DPPH\;Scavenging\;Activity(\%)=\frac{A_{c}-A_{s}}{A_{c}}\times 100$$

A calibration curve correlating DPPH scavenging activity (%) with Trolox concentration (μg/mL) was generated using standard solutions of Trolox within the concentration range of 1.0 to 25 μg Trolox/mL [38]. The antioxidant activity of fruit powder was calculated and expressed as μM Trolox equivalents (TE) per gram of dry weight (d.w.).

#### 2.4.7. Assessment of Antioxidant Activity by Ferric Reducing Antioxidant Power (FRAP) Assay

The ferric reducing antioxidant power (FRAP) assay was used to determine the total antioxidant potential of the samples. This method assesses the ability of antioxidant constituents in ethanol extracts to reduce ferric ions (Fe^3+^) from a colorless tripyridyltriazine complex to ferrous ions (Fe^2+^) in an acidic medium. This redox reaction, driven by electron donation from antioxidant species, results in the formation of an intense blue-colored complex with tripyridyltriazine (TPTZ), which exhibits maximum absorption at 593 nm [39]. The FRAP working solution was prepared by mixing 100 mL of acetate buffer (pH 3.6), 10 mL of a 10 mM TPTZ solution in 40 mM HCl, and 10 mL of a 20 mM FeCl_3_·6H_2_O solution. Prior to measurement, the initial alcoholic extracts of the fruit powders were diluted with distilled water at a 1:50 (*v*/*v*) ratio. For the assay, 0.5 mL of the diluted extracts was allowed to react with 2.5 mL of the FRAP working solution at 37 °C for 30 min. The resulting absorbance was measured at 593 nm, against a blank solution prepared without the sample under identical conditions. The antioxidant capacity was quantified as µM Fe^2+^ equivalents per gram of dry weight, based on a calibration curve generated with FeSO_4_·7H_2_O standard solutions in the concentration range of 0.05 to 0.5 µM Fe^2+^ equivalents/mL. Each analysis was conducted in three independent replicates.

### 2.5. Proximate Composition and Energy Value Evaluation of Salami Formulations

The proximate analysis of the salami formulations was performed following appropriate methods recommended by AOAC [40], such as AOAC 950.46 for moisture, AOAC 973.48 for protein, AOAC 960.39 for fat, AOAC 937.09 for NaCl, and AOAC 999.11 for ash, respectively. The carbohydrate content was calculated by subtracting from 100 the sum of the protein, ash, lipid, NaCl, and moisture content. The energy values for each salami formula, expressed in kcal/100 g, were calculated taking into account their carbohydrate, fat, and protein content and using the specific energy factors for protein (4 calories per gram), for fat (9 calories per gram), and carbohydrates (4 calories per gram) [41]. Each analysis was conducted in three independent replicates.

### 2.6. Assessing the Progression of Lipid Oxidation

Lipid oxidation in salami samples, stored under refrigerated conditions (4 °C) for 0, 15, and 30 days, was assessed by measuring specific chemical indices. These included peroxide value (PV), para-anisidine value (pAV), and thiobarbituric acid (TBA) value. Additionally, inhibition of oxidation (IO) and total oxidation (TOTOX) values were calculated. All analyses were conducted in three independent replicates.

#### 2.6.1. Peroxid Value (PV)

To assess the extent of primary lipid oxidation in salami samples, peroxide value (PV) was determined. This indicator quantifies the concentration of hydroperoxides generated during the initial oxidation stage. The analysis followed the iodometric method [42] and was performed on the lipids extracted from salami samples with a mixture of chloroform and methanol (2:1, *v*/*v*) following the procedure described by Seo et al. [43]. PV was reported as milliequivalents of active oxygen per kilogram of lipid.

#### 2.6.2. Para-Anisidine Value (pAV)

To assess the extent of secondary lipid oxidation, the quantification of aldehydic compounds, as secondary oxidation products, was performed by means of the para-anisidine value (pAV). This analysis was performed following the methodology detailed in the International Organization for Standardization (ISO) protocol 6885:2008 [44]. The basic principle of this test involves the nucleophilic addition of the amine group of para-anisidine to the electrophilic carbonyl group of aldehydes, resulting in a Schiff base that exhibits maximum absorption at a wavelength of 350 nm. For the analysis, a precisely weighed 2-g aliquot of extracted fat according to Seo et al. [43] was dissolved in 25 mL of isooctane, and its baseline absorbance was spectrophotometrically determined at 350 nm using isooctane as the reference (Abs_I_). Subsequently, a 5 mL portion of this initial solution was reacted with 1 mL of a para-anisidine reagent (0.25% *w*/*v* in glacial acetic acid) in a separate vessel. Following a 10 min reaction period, the absorbance of this mixture was measured at 350 nm against a control comprising 5 mL of isooctane and 1 mL of the anisidine reagent, (Abs_II_). The pAV was then computed using the relationship presented in Equation (2):

(2) $$pAV=25\times \frac{1.2\times {Abs}_{II}-{Abs}_{I}}{m}$$

where Abs_I_ and Abs_II_ represent the absorbance of the fat sample in isooctane and in isooctane with para-anisidine solution, respectively; m—mass of the fat sample (g).

#### 2.6.3. Inhibition of Oxidation (IO)

The inhibition of lipid oxidation (IO), achieved by adding either sodium nitrite or fruit powder, was quantified following the method by Mariod et al. [45]. This assessment relied on comparing the peroxide value (PV) increase in samples after 15 and 30 days of storage, relative to day 0, against the PV increase observed in the control sample over the same storage period. The calculation was performed using the relationship detailed in Equation (3).

(3) $$IO(\%)=(1\;-\;\frac{increase\;in\;PV\;of\;sample}{increase\;in\;PV\;of\;control})\times 100$$

#### 2.6.4. TOTOX Value

Based on the findings of a previous study [2], the simultaneous assessment of both primary and secondary markers of lipid oxidation is considered more effective for a comprehensive understanding of oxidative deterioration. This dual approach provides insight into the overall extent of oxidation by including the accumulation of secondary oxidation products alongside the oxidative status indicated by primary species. The TOTOX value was calculated by combining the peroxide value (PV) and the para-anisidine value (pAV), according to Equation (4) [46].

(4) TOTOX value = pAV + 2 × PV

#### 2.6.5. Thiobarbituric Acid (TBA) Value

To quantify the secondary products of lipid oxidation, particularly malondialdehyde (MDA), the thiobarbituric acid (TBA) test was performed [47]. This colorimetric method is based on the principle that MDA reacts with TBA under heated acidic conditions to produce a colored complex, the concentration of which is proportional to the MDA content. To this end, 5 g of the minced salami sample was homogenized by shaking for 5 min in 20 mL of 5% trichloroacetic acid solution (*w*/*v*). The resulting homogenate was then centrifuged at 12,000 rpm for 10 min to obtain a clear supernatant. Subsequently, a 4 mL aliquot of the supernatant was combined with an equal volume (4 mL) of a 0.02 M aqueous TBA solution. The resulting mixture was then heated for 60 min at 100 °C in a water bath to facilitate color development. Following incubation and cooling to ambient temperature, the absorbance of the resulting solution was measured at 532 nm using a Specord 205 spectrophotometer (Analytik Jena Inc., Jena, Germany). A reagent blank, devoid of the fat sample, was used as the reference for these spectrophotometric determinations. A fat-free sample prepared under the same operating conditions was used as a reference. The MDA concentration in the samples was determined by interpolating the absorbance values against a standard calibration curve prepared with MDA solutions ranging from 10 to 50 µg/mL. TBA values were expressed as mg MDA per kg of sample and represent the average of three independent measurements.

### 2.7. Statistical Analysis

The data obtained are reported as mean values, followed by the standard deviation (SD). A one-way analysis of variance (ANOVA) was used to evaluate the statistical significance of the differences between the fruit powders, as well as between the salami samples, in response to the storage period and the supplementation variant. Levene’s test was utilized to confirm homogeneity of variances. Following this, post hoc analysis was conducted via Tukey’s test, and all differences were considered statistically significant at *p* < 0.05.

## 3. Results and Discussion

### 3.1. Phytochemical Profile and Antioxidant Activity of Fruit Powder

Table 3 presents the phytochemical profiles of the investigated fruit powders, blackcurrant (BCP), lingonberry (LP), and sour cherry (SCP), including total phenolic, flavonoid, and monomeric anthocyanin contents, ascorbic acid, and antioxidant activity measured via ferric reducing antioxidant power (FRAP) and DPPH radical scavenging assays.

#### 3.1.1. Total Phenolic Content (TPC)

BCP exhibited the highest TPC, with values 54.35% and 28.00% greater than SCP and LP, respectively. This is consistent with Kim [48], who reported a high TPC of approximately 710.33 mg GAE/100 g fresh weight (f.w.) in fresh blackcurrants, as well as Sadowska et al. [49], who found blackcurrant powders ranging from 613.7 to 1577.2 mg GAE/100 g d.w., influenced by drying methods applied. It is worth noting that the TPC value determined for BCP in this study aligns with a previously reported range [49]. A slightly higher TPC value for blackcurrant powder was also documented, reaching 19.84 mg GAE/g d.w. [50]. For LP, the TPC values align with Daukšienė et al. [32], who reported a wide range (6.75–27.19 mg/g d.w.) in dried fruit depending on the preparation method, highlighting the impact of drying techniques on phenolic compounds retention.

Meanwhile, freeze-dried sour cherry powders exhibited the lowest total phenolic content (TPC), ranging from 1.87 to 6.80 mg GAE/g [51], with broader variability reported among cultivars (96.56–268.98 mg GAE/100 g f.w.) [52]. Additionally, depending on the extraction parameters applied, ultrasound-assisted extraction methods have yielded TPC values ranging from 8.45 to 17.50 mg GAE/g d.w. [53].

#### 3.1.2. Total Flavonoid Content (TFC)

As presented in Table 3, BCP demonstrated the highest flavonoid content, exceeding that of SCP by 43.92% and LP by 14.24%. Previous research conducted by Yaman [54] on various sour cherry genotypes reported total flavonoid content (TFC) values ranging from 0.68 to 1.35 mg QE/g f.w. When adjusted for moisture content, our results are in good agreement with these previously reported ranges. Borowiec et al. [50] documented a TFC of 8.28 mg QE/g d.w. in blackcurrant powder, which aligns well with the findings of the present study. In contrast, Blejan et al. [55] reported a considerably higher value (24.43 mg QE/g d.w.) in lyophilized blackcurrant pomace. This discrepancy can be attributed to the higher concentration of polyphenols in the skins and seeds retained in pomace. Moreover, freeze-drying, as applied to the pomace, is known to better preserve flavonoid compounds compared with conventional techniques such as air- or oven-drying.

#### 3.1.3. Total Monomeric Anthocyanin Content (TMA)

The TMA in BCP was significantly higher (859.04 mg C3G/100 g d.w.) than in SCP (509.73 mg) and LP (312.58 mg), consistent with Lee et al. [56], who reported similar values for blackcurrants (813.60 mg/100 g d.w.). Borowiec et al. [50] documented lower TMA in blackcurrant powders (520 mg/100 g d.w.), possibly due to differences in source material or drying methods. For SCP, the TMA values align well with those reported by Sokół-Łętowska et al. [52], 83.24 mg C3G/100 g f.w., when adjusted to dry weight equivalents.

Lingonberry TMA levels (306–396 mg C3G/100 g dry weight) reported by Ozola and Kampuse [57], as well as lower fresh weight levels documented by Dróżdż et al. [58] and Koponen et al. [59], support the variability observed and emphasize the influence of origin and processing. Consistent with Ozola and Kampuse [57], TMA content in dried lingonberry byproducts ranged from 306 to 396 mg C3G/100 g d.w., varying by drying method. Dróżdż et al. [58] reported 33–47 mg/100 g f.w. in Polish wild lingonberries, while Koponen et al. [59] found 77.5 mg/100 g f.w. in berries grown in Finland. Adjusted to dry weight, these values align with our findings.

#### 3.1.4. L-Ascorbic Acid Content (AsAc)

BCP demonstrated remarkably high ascorbic acid content (621.09 mg/100 g dry weight), being approximately 9.63 and 13.53 times richer than LP (64.52 mg) and SCP (45.91 mg), respectively. These findings align with Sadovska et al. [49], who documented ascorbic acid contents from 445.2 to 861.2 mg/100 g d.w. in blackcurrant powders depending on drying conditions. The value for the convection drying method (620.50 mg/100 g d.w.) was comparable to our data. Urbonaviciene et al. [60] reported ascorbic acid levels in lingonberries ranging from 8.8 to 9.6 mg/100 g f.w., while Wojdyło et al. [61] found values between 5.5 and 22.1 mg/100 g f.w. in sour cherries; both are consistent with our results after moisture adjustment.

#### 3.1.5. Antioxidant Activities (FRAP and DPPH Assays)

Antioxidant capacity, evaluated via ferric reducing antioxidant power (FRAP) and DPPH radical scavenging assays (Table 3), revealed the highest electron-donating and radical-quenching activities in BCP. FRAP values for BCP were higher than those of SCP by 42.37% and LP by 13.21%, while DPPH scavenging followed a similar pattern, with BCP significantly (*p* < 0.05) exceeding SCP and LP by 39.30% and 10.33%, respectively. BCP consistently demonstrated the highest antioxidant activity in both assays, which can be attributed to its elevated levels of total phenolics and flavonoids. These compounds not only act as electron or hydrogen donors but also contribute to the formation of stable radical intermediates, an essential mechanism in mitigating lipid oxidation in fat-rich food matrices. 

Kim [48] observed lower DPPH free radical scavenging activity in blackcurrants (265.58 μM TE/g d.w.). Similarly, Sokół-Łętowska et al. [52] reported FRAP antioxidant levels in various sour cherry cultivars that closely match our findings. Daukšienė et al. [32] documented FRAP values for dried lingonberries between 164.5 and 550.67 µmol/g d.w., aligning well with our data. These findings reinforce that the antioxidant effects of fruit powder polyphenols arise from their molecular structure, enabling efficient electron donation and the stabilization of free radicals. These strong antioxidant properties underscore the potential of fruit powders as natural preservatives, positioning them as promising alternatives to synthetic additives in meat products.

### 3.2. The Proximate Composition of Salami Formulations

The proximate composition and energy value for both SI and SII salami formulations are shown in Table 4.

The values for the proximate composition fall within the ranges established for cooked and scalded salami by Order 210/2006 [62]. The moisture content of both SI and SII salami formulations was affected by the type and dose of fruit powder added, as well as the ingredients and the heat treatment applied (cooking or scalding). The moisture content of SI samples ranged from 60.27 g/100 g to 61.24 g/100 g, while that of SII samples varied between 62.31 g/100 g and 63.27 g/100 g. For both salami types, the highest moisture content values were recorded in the control samples: SI-C (61.24 g/100 g) and SI-CN (61.21 g/100 g), SII-C (63.27 g/100 g), and SII-CN (63.28 g/100 g). The addition of fruit powders (SCP, BCP, and LP) resulted in a gradual and statistically significant decrease (*p* < 0.05) in moisture content for both salami types. The reduction in moisture content is mainly due to the low moisture content of the fruit powder (below 5%). This observation aligns with Mahapatra et al. [63], who reported reduced moisture content in heat-treated meatballs fortified with fruit and fruit byproduct powders compared with controls. Fu et al. [64] reported that increasing levels of cherry powder in sausages led to greater moisture loss during 30 days of refrigerated storage. This suggests that higher concentrations of cherry powder may adversely impact the product’s water-holding capacity. One possible reason is that the cherry powder interferes with protein–water interactions. Polyphenols present in the powder may bind to muscle proteins, displacing water molecules that would otherwise form hydrogen bonds and contribute to water retention. This substitution diminishes the ability of proteins to retain water in the meat matrix.

The protein contribution from the fruit powder in the salami formulations doesn’t significantly impact the overall protein values in SI and SII salami types. The protein content in the SI salami formulation exhibited a slight reduction, decreasing from 11.52 g/100 g in the SI-CN sample to 11.31 g/100 g in the SI-SCP300 formulas. Similarly, the SII salami formulation also showed a decrease in protein content, falling from 12.65 g/100 g in the SII-CN formula to 12.43 g/100 g in the SII-SCP300 sample. Compared to the protein content in the control samples, both SI and SII salami formulations exhibited a minor reduction in protein content as the concentration of added fruit powder increased. Our observations are consistent with the findings reported by Cocan et al. [28], who documented a reduction in protein content following the incorporation of bell pepper byproduct powder into pork sausages.

Supplementation of salami with varying doses of fruit powder significantly (*p* < 0.05) affected lipid content. As the amount of added powder increased, a gradual decrease in lipid content was observed. In SI salami, the lipid content declined from 22.02 g/100 g in the SI-C sample to 21.43 g/100 g in the SI-SCP300 formulation. Similarly, in SII, it decreased from 19.53 g/100 g in the SII-CN sample to 19.02 g/100 g in the SII-SCP300 formulation. The SI samples consistently exhibited lipid content approximately 2.5 g/100 g higher than that of SII, a difference attributed to the specific manufacturing recipe, which incorporated a greater amount of fat in the SI formulations. A gradual reduction in lipid content with increased fruit powder supplementation was also reported by Mahapatra et al. [63], who observed a decrease in lipid levels in meatballs fortified with starfruit and guava powders. Similar results were obtained by Zaini et al. [65], where the low lipid content of banana peel powder contributed to reducing the lipid levels in chicken sausages.

The ash content increased proportionally with the quantity of SCP, BCP, and LP integrated into the salami formulas. This can be attributed to the minerals and vitamins supplied by the fruit powders. Ash content in the smoked and cooked (SI) salami ranged from 1.85 to 2.19 g/100 g, while the smoked and scalded (SII) salami showed values between 1.92 and 2.28 g/100 g. The highest levels were recorded in SI-SCP300 (2.19 g/100 g) and SII-SCP300 (2.28 g/100 g). A similar trend in ash content was observed in the investigation conducted by Zaini et al. [65], who noted a progressive increase in the ash content of analyzed chicken sausage samples (3.04–5.77%) with incremental additions of banana peel powder. Furthermore, Lopez-Vargas et al. [66] reported a comparable pattern, indicating a 2.5–5% increase in the ash content of the formulated pork burger samples following the inclusion of passion fruit peel powder.

The NaCl content in the analyzed SI and SII salami formulations showed no significant differences (*p* > 0.05) in response to fruit powder incorporation. Values ranged from 2.19–2.24 g/100 g for SI and 2.10–2.15 g/100 g for SII. Our findings align with the values documented in the literature, particularly those by Zanardi et al. [67], who reported a content of 1–2.5% for Italian salami.

Carbohydrate content gradually increased in both types of salami with incremental SCP, BCP, and LP supplementation. In SI samples, values ranged from 1.19 to 2.58 g/100 g, while in SII formulations, they ranged between 0.48 and 1.81 g/100 g.

The inclusion of fruit powder in the salami formulations did not significantly impact their energy value. However, a modest reduction in energy value was evident across both salami types, correlating with an increase in the level of fruit powder incorporation. A similar trend has been documented in other investigations, with an increase in the proportion of tomato processing byproducts incorporated into pork sausages [27].

### 3.3. Progression of Lipid Oxidation

Several lipid oxidation indices were evaluated to monitor the progression of oxidation in salami samples stored for 0, 15, and 30 days under refrigeration (4 °C). The peroxide value indicated primary oxidation, while the para-anisidine value and thiobarbituric acid reactive substances reflected secondary oxidation. Inhibition of lipid oxidation and the TOTOX value complemented the assessment of the oxidative status of the investigated products.

#### 3.3.1. Peroxide Value

Chemical spoilage in meat products during processing and storage primarily results from lipid degradation by autooxidation. The initial stage of lipid oxidation leads to the formation of hydroperoxide or the loss of polyunsaturated fatty acids. Measurement of lipid hydroperoxide formation, commonly expressed as the peroxide value, has long served as a key indicator of primary oxidation compounds in meat and meat products [2].

**Figure 2:**
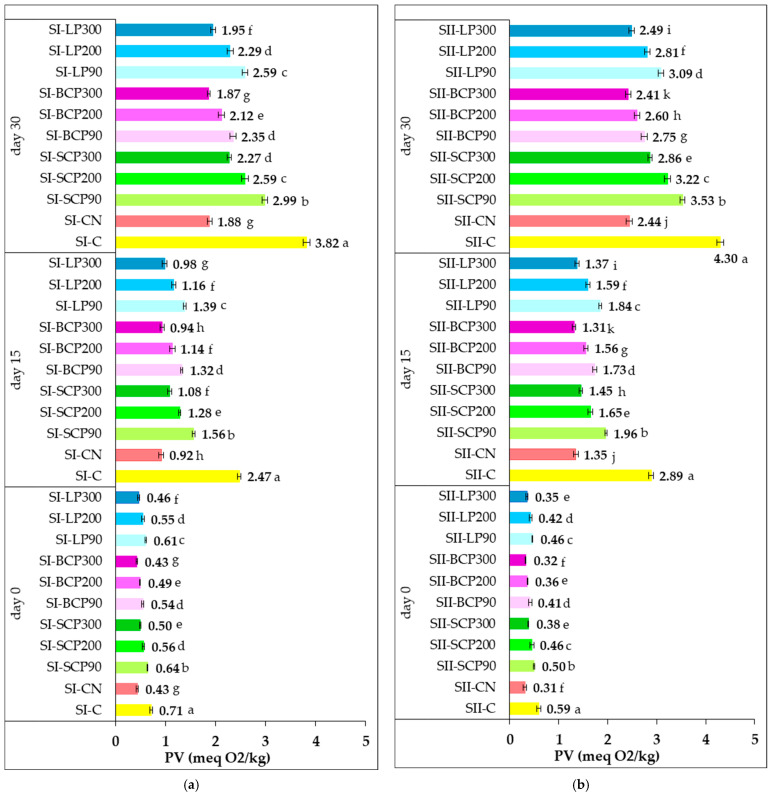
Changes in peroxide value (PV) during cold storage of smoked and cooked salami (SI) (**a**) and smoked and scalded salami (SII) (**b**), following supplementation with sodium nitrite and fruit powder. SI-C, SII-C: Nitrite-free SI and SII salami; SI-CN, SII-CN: SI and SII salami with added sodium nitrite; SI-SCP90, SI-SCP200, SI-SCP300, SII-SCP90, SII-SCP200, SII-SCP300: Nitrite-free SI and SII salami with sour cherry powder added to provide a TPC of 90, 200 and 300 mg GAE/kg of meat; SI-BCP90, SI-BCP200, SI-BCP300, SII-BCP90, SII-BCP200, SII-BCP300: Nitrite-free SI and SII salami with blackcurrant powder added to provide a TPC of 90, 200 and 300 mg GAE/kg of meat; SI-LP90, SI-LP200, SI-LP300, SII-LP90, SII-LP200, SII-LP300: Nitrite-free SI and SII salami with lingonberry powder added to provide a TPC of 90, 200 and 300 mg GAE/kg of meat. Results are expressed as the mean ± standard deviation (SD) of three independent analyses. Means labeled with different letters within the same storage period are significantly different (one-way ANOVA, *p* < 0.05).

Figure 2 illustrates the effect of supplementing salami formulations with sodium nitrite and fruit powder on peroxide value (PV) during cold storage for 0, 15, and 30 days.

Immediately following processing (day 0), PV measured were low, ranging from 0.31 to 0.59 meq O_2_/kg in SI samples and from 0.43 to 0.71 meq O_2_/kg in SII samples. A low PV value is an indicator of freshness and quality, indicating that the product has been manufactured, stored, and handled properly [68].

The results indicate that incorporating fruit powders into salami significantly enhances oxidative stability during processing. This beneficial effect is both dose-dependent and species-dependent, as higher concentrations of fruit powder more effectively inhibit oxidative processes and improve lipid stability. This is evidenced by the lower peroxide values observed in samples enriched with higher doses (e.g., BCP300, LP300, SCP300), as well as by the superior oxidative protection provided by BCP compared with LP and SCP.

The differences in PV on day 0 for SI and SII are attributed to their distinct processing conditions. The SII salami, heat-treated at 72 °C under 80% relative humidity, exhibited enhanced oxidative stability and lower PV compared with the SI samples, which underwent heat treatment at the same temperature but under low relative humidity (5%). Under such conditions, lipid oxidation is primarily driven by elevated temperature and increased oxygen availability. For SI samples, low relative humidity increases lipid exposure to oxygen by limiting the protective moisture layer, thereby accelerating oxidation. Additionally, surface drying can concentrate pro-oxidants such as metal ions, further promoting peroxidation [69]. In contrast, the higher relative humidity used for SII creates a water barrier that reduces oxygen diffusion to lipid surfaces, thus slowing the oxidation rate [70]. These findings underscore the importance of precisely controlling relative humidity and temperature during meat processing to optimize lipid stability and overall product quality.

During cold storage, a general increase in PV was observed, indicating progressive lipid oxidation. Extending the storage period from 15 to 30 days significantly intensified primary oxidation processes in the samples. Significant differences (*p* < 0.05) in PV were recorded between the control and the samples supplemented with nitrite or fruit powders in both SI and SII formulations. These variations are linked to the antioxidant effects of SCP, BCP, and LP powders added at three dosage levels. Samples supplemented with higher levels of fruit-derived total phenolics showed lower PV at both 15 and 30 days of storage, reflecting enhanced oxidative stability. When normalized for equivalent total phenolic content (TPC), the powders ranked in effectiveness as follows: BCP > LP > SCP.

After 30 days, SI samples (60.27–61.24% moisture) exhibited lower PV values compared to SII samples (62.31–63.27%). The greater lipid peroxidation in SII, despite initially lower oxidation, suggests that its higher moisture content facilitated oxidative degradation over extended storage.

At the end of storage, salami samples supplemented with fruit powders exhibited PVs ranging from 1.87 to 2.99 meq O_2_/kg for SI and from 2.41 to 3.53 meq O_2_/kg for SII. In contrast, the negative control samples (SI-C and SII-C) showed higher PVs of 3.82 and 4.30 meq O_2_/kg, respectively. Samples containing nitrite (SI-CN and SII-CN) demonstrated a strong antioxidative effect, with PVs of 1.88 and 2.44 meq O_2_/kg, respectively. Among the fruit powders tested, BCP was the most effective in limiting hydroperoxide formation.

At all storage points, BCP added to both SI and SII salami formulations at 300 mg GAE/kg of processed meat exhibited the strongest inhibition of primary lipid oxidation, as evidenced by consistently low PV values. No significant differences in PV were observed between SI-BCP300 and SI-CN, or between SII-BCP300 and SII-CN samples, indicating that BCP’s inhibitory effect was comparable to that of nitrite.

After 30 days of cold storage, the peroxide values of lipids extracted from the fruit-powder-enriched salami samples remained below 5 meq O_2_/kg. This level is characteristic of non-rancid fats, which typically show low PV, often under 5 meq O_2_/kg [71]. In general, peroxide values between 0 and 6 suggest that oxidation has not significantly progressed, while values from 7 to 10 may indicate the initial stages of rancidity. A PV exceeding 10 is generally associated with advanced lipid oxidation, rendering the fat unsuitable for consumption [71]. Previous research by Yi et al. [68] suggests that the upper limit for acceptable PVs in oils and fats lies between 5 and 10 meq O_2_/kg. In line with these findings, the PVs observed in this study remained within the maximum allowable limit of 10 milliequivalents of active oxygen per kilogram of fat, as defined by the Codex Standard for Named Animal Fats (CODEX STAN 211–1999) [72]. The inhibition of primary oxidation in salami through the incorporation of fruit powders aligns with existing literature, which demonstrates that adding various fruit types and fruit-derived products to meat effectively prevents lipid oxidation and significantly extends shelf life [73,74]. Similarly, other studies have reported a decrease in the PV of soybean oil with increasing levels of natural antioxidants [75]. These results support the use of fruit powders as functional ingredients with preservative properties during meat processing and storage.

#### 3.3.2. Inhibition of Oxidation

Table 5 shows the changes in inhibition of lipid oxidation (IO) during cold storage of SI and SII formulations supplemented with sodium nitrite and fruit powder.

The IO (%) was calculated from PV to assess early lipid oxidation and reflects the anti-peroxidative effect of fruit powders in SI and SII formulations [76]. In all salami samples enriched with fruit powders, a clear inhibitory effect on lipid oxidation was observed during refrigerated storage at both 15 and 30 days. The inhibition of lipid oxidation was dose-dependent across both SI and SII formulations. Among the tested powders, BCP showed the strongest inhibitory effect, followed by LP and SCP, sustained through to day 30. On day 15, the IO of BCP300 in SI (71.10%) was slightly below that of nitrite (72.77%), while in SII, BCP300 outperformed nitrite (57.08% vs. 54.83%). By day 30, the inhibitory effect of both nitrite and fruit powders declined. In SI, BCP300 matched nitrite’s IO (53.82% vs. 53.59%), while in SII it remained slightly higher (43.66% vs. 42.43%). LP300 also demonstrated comparable effectiveness, exhibiting a slightly lower IO than nitrite in SI (52.22%) and an almost identical value in SII (42.38%). SCP exhibited a weaker inhibitory effect compared to BCP and LP, with IO values for SCP300 approximately 20–22% lower than nitrite after 30 days. Both BCP and LP maintained their antioxidant activity throughout prolonged storage, especially at doses to provide 300 mg GAE/kg, making them promising natural alternatives to nitrite in processed meat

#### 3.3.3. Para-Anisidine Value (pAV)

**Figure 3:**
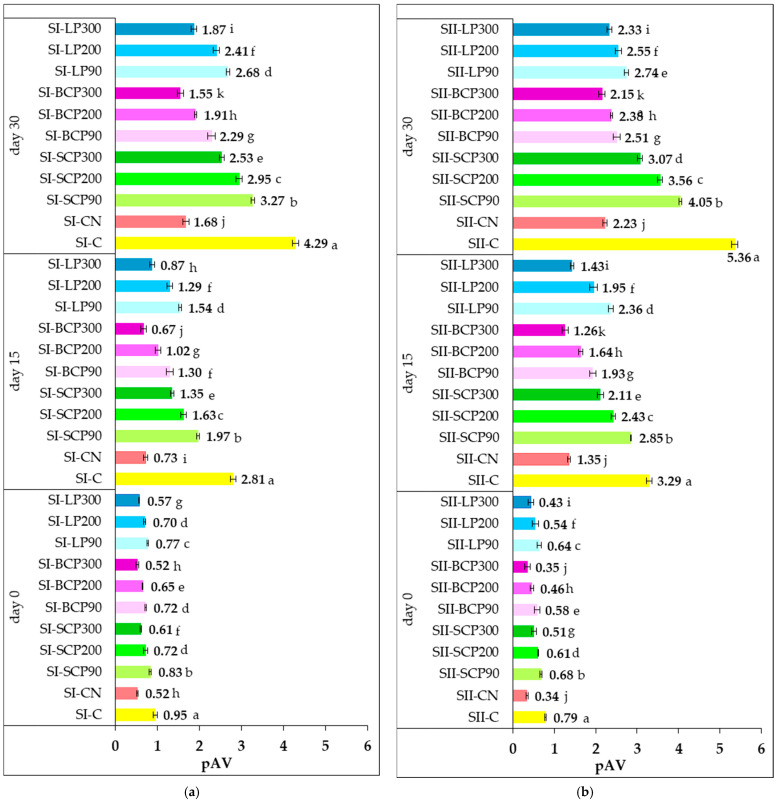
Impact of sodium nitrite and fruit powder supplementation on para-anisidine value (pAV) during cold storage of smoked and cooked salami (SI) (**a**) and smoked and scalded salami (SII) (**b**). SI-C, SII-C: Nitrite-free SI and SII salami; SI-CN, SII-CN: SI and SII salami with added sodium nitrite; SI-SCP90, SI-SCP200, SI-SCP300, SII-SCP90, SII-SCP200, SII-SCP300: Nitrite-free SI and SII salami with sour cherry powder added to provide a TPC of 90, 200 and 300 mg GAE/kg of meat; SI-BCP90, SI-BCP200, SI-BCP300, SII-BCP90, SII-BCP200, SII-BCP300: Nitrite-free SI and SII salami with blackcurrant powder added to provide a TPC of 90, 200 and 300 mg GAE/kg of meat; SI-LP90, SI-LP200, SI-LP300, SII-LP90, SII-LP200, SII-LP300: Nitrite-free SI and SII salami with lingonberry powder added to provide a TPC of 90, 200 and 300 mg GAE/kg of meat. Results are expressed as the mean ± standard deviation (SD) of three independent analyses. Means labeled with different letters within the same storage period are significantly different (one-way ANOVA, *p* < 0.05).

Prolonged cold storage of meat products can lead to lipid peroxidation, compromising quality. While peroxide value (PV) reflects early oxidation, it does not account for later-stage changes [77]. Para-anisidine value (pAV) is a reliable indicator of secondary oxidation, measuring aldehydes like 2-alkenals and 2,4-alkadienals formed from peroxide breakdown during advanced oxidative stages [2]. Figure 3 illustrates the pAV values measured on days 0, 15, and 30 of refrigerated storage for the various salami formulations.

The changes in pAV reflect the replacement of sodium nitrite with fruit powders, added at levels corresponding to total phenolic contents of 90, 200, and 300 mg GAE/kg of processed meat. The data show that SCP, BCP, and LP effectively suppressed secondary lipid oxidation in both SI and SII samples throughout storage when compared with the positive and negative controls. On day 0, pAV levels were low across all formulations containing either sodium nitrite or fruit powder, although they increased over time. No significant differences (*p* > 0.05) were observed on day 0 between SI-BCP300 and SII-BCP300 and the respective nitrite-containing controls (SI-CN and SII-CN), indicating that BCP at a level providing 300 mg GAE/kg of processed meat showed a comparable protective effect against secondary oxidation during processing.

Secondary lipid oxidation followed a similar trend to primary oxidation, with higher oxidation rates observed under the dry conditions (5% relative humidity) characteristic of the SI processing. This was probably due to the absence of a protective water barrier. Conversely, the SII samples exhibited lower levels of secondary oxidation, which may be attributed to heat treatment under high humidity (80%). This condition has probably favored the formation of a physical barrier or surface film around the lipid molecules, thereby reducing oxygen diffusion, diluting pro-oxidant catalysts, and limiting the movement of water-soluble radicals into the lipid phase [69,70].

Throughout cold storage, the addition of fruit powders at all tested levels significantly reduced pAV compared with the SI-C and SII-C controls. The inhibitory effect on secondary lipid oxidation intensified with increasing TPC levels supplied by the fruit powders. Significant differences (*p* < 0.05) were observed in the pAV among salami samples enriched with different fruit powders and at varying inclusion levels. Among these, BCP supplementation demonstrated the strongest inhibition of secondary lipid oxidation during processing and after 15 and 30 days of storage. Accordingly, the lowest pAV was recorded in BCP-enriched samples, followed by those containing LP and SCP.

After 30 days of storage, the pAV of the fruit-powder-enriched salami formulas ranged from 1.55 to 3.27 for SI and 2.15 to 4.05 for SII. In contrast, the pAV of the negative control samples (SI-C and SII-C) was higher, at 4.29 and 5.36, respectively. In the positive control samples (SI-CN and SII-CN), a strong inhibitory effect was observed due to the addition of nitrite, quantified by low pAV values of 1.68 for SI-CN and 2.23 for SII-CN.

The results obtained after cold storage suggest that the incorporated fruit powders exhibited a specific inhibitory effect on secondary lipid oxidation processes, dependent on the type and dose, thereby having a positive impact on oxidative stability. Throughout cold storage, BCP300 showed the strongest inhibitory potential on secondary oxidation processes, both in SI and II, higher than that of sodium nitrite. At the end of storage, pAV of 1.55 was recorded for the SI-BCP300 and 2.15 for the SII-BCP300 sample. These values were lower than those observed in the positive control samples, SI-CN (1.68) and SII-CN (2.23).

The decomposition of hydroperoxides into secondary lipid oxidation products after 15 and 30 days of storage was more pronounced in the SII formulations compared with the SI samples, as indicated by the higher pAV. The higher moisture content in the SII samples probably accelerated secondary oxidation during prolonged storage by increasing the mobility of reactive species and facilitating degradation reactions over time.

Overall, both during processing and storage, pAV decreased progressively with increasing TPC supplied by the incorporated fruit powders. This trend aligns with findings from previous studies on smoked and scalded sausages supplemented with powders derived from vegetable processing byproducts [27,28]. The low pAV values indicate the effectiveness of fruit powders in slowing the formation of secondary oxidation products, suggesting that the fats are less prone to rancidity.

Although no official regulatory limits for p-anisidine value (pAV) in edible oils and fats currently exist, a threshold of 10 is commonly referenced, with values below 4 recommended to ensure high quality [78]. In addition, the GOED Voluntary Monograph [79] considers a pAV below 20 as the acceptable limit for product quality.

#### 3.3.4. TOTOX Value

**Figure 4:**
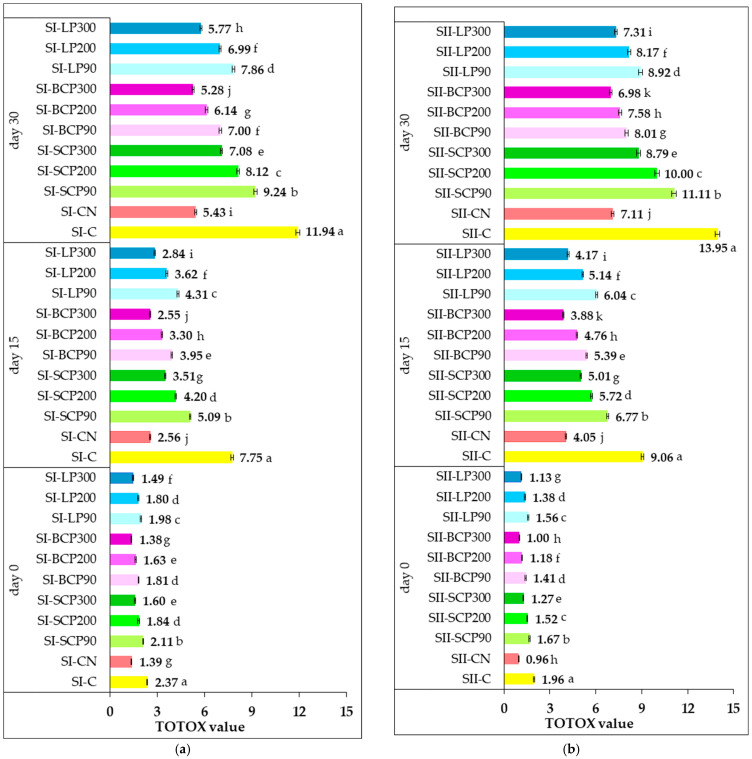
Effect of sodium nitrite and fruit powder supplementation on TOTOX value during cold storage of smoked and cooked salami (SI) (**a**) and smoked and scalded salami (SII) (**b**). SI-C, SII-C: Nitrite-free SI and SI salami; SI-CN, SII-CN: SI and SII salami with added sodium nitrite; SI-SCP90, SI-SCP200, SI-SCP300, SII-SCP90, SII-SCP200, SII-SCP300: Nitrite-free SI and SII salami with sour cherry powder added to provide a TPC of 90, 200 and 300 mg GAE/kg of meat; SI-BCP90, SI-BCP200, SI-BCP300, SII-BCP90, SII-BCP200, SII-BCP300: Nitrite-free SI and SII salami with blackcurrant powder added to provide a TPC of 90, 200 and 300 mg GAE/kg of meat; SI-LP90, SI-LP200, SI-LP300, SII-LP90, SII-LP200, SII-LP300: Nitrite-free SI and SII salami with lingonberry powder added to provide a TPC of 90, 200 and 300 mg GAE/kg of meat. Results are expressed as the mean ± standard deviation (SD) of three independent analyses. Means labeled with different letters within the same storage period are significantly different (one-way ANOVA, *p* < 0.05).

Lipid oxidation is a gradual process, with an increase in PV being followed by an increase in pAV. Evaluating a singular class of oxidation products is generally insufficient for a comprehensive and accurate assessment of a sample’s oxidative status. A more robust approach involves the concurrent analysis of both primary and secondary lipid oxidation markers [2]. This dual measurement strategy provides insights into the complete oxidative trajectory. Consequently, despite its classification as a derivative indicator rather than a direct analytical technique, TOTOX has been proposed as a tool for quantifying the overall oxidation of fat samples. Figure 4 shows the effect of salami formulas supplementation with fruit powder and nitrite on TOTOX value during cold storage for 15 and 30 days.

Generally, fresh meat products are characterized by low TOTOX values. As shown, the TOTOX for salami samples on day 0 of storage showed low values in the range 1.38–2.37 for SI and 0.96–1.96 for SII. The low TOTOX value could be an indication that the salami samples were properly manufactured and that they would not easily go rancid when properly stored in refrigeration conditions.

The results demonstrate oxidative changes occurred during 15 and 30 days of storage, as evidenced by increases in TOTOX values. A closer look at the results reveals that control samples (SI-C and SII-C) had significantly higher TOTOX values (*p* < 0.05) after both 15 and 30 days of storage, in comparison with both the positive control samples and the fruit-powder-enriched salami samples.

After 30 days of storage, the SI-C and SII-C control samples reached TOTOX values of 11.94 and 13.95, respectively. In contrast, incorporating sodium nitrite resulted in lower TOTOX values of 5.43 for the SI-CN sample and 7.11 for the SII-CN sample. All incorporated fruit powders significantly (*p* < 0.05) reduced lipid oxidative deterioration. Specifically, salami samples supplemented with SCP, BCP, and LP recorded significantly lower (*p* < 0.05) TOTOX values than those measured for the SI-C and SII-C on both day 15 and day 30 of storage. This beneficial effect can be attributed to the phenolic compounds provided by the incorporated fruit powders. Natural antioxidants generally delay lipid oxidation by donating hydrogen atoms or chelating metal ions. Berries, in particular, are rich in polyphenols—such as phenolic acids and flavonoids—which can effectively scavenge free radicals, thereby inhibiting lipid oxidation. Various plant-derived sources, including blackcurrants, blueberries, apples, aronia, and elderberry powders, have been documented as effective antioxidants in pork patties, demonstrating differing capacities to neutralize free radicals and reduce both primary and secondary lipid oxidation [80].

In our study, BCP showed a higher radical scavenging ability than that of LP and SCP, which was proven to converge with their high ability to inhibit lipid oxidation.

It can be stated that the addition of sodium nitrite and fruit powder, respectively, significantly increased the oxidative stability of the salami formulations both during processing and storage, compared with the control (*p* < 0.05). It was observed that TOTOX values decreased with increasing fruit powder dose, with the lowest values recorded in salami samples (both SI and SII formulations) enriched with a dose of fruit powder ensuring a TPC of 90 mg GAE/kg of processed meat.

The fruit species from which the fruit powder was derived also had an effect on the TOTOX values obtained. The lowest TOTOX values were recorded for salami with added SCP, followed by samples fortified with LP and BCP.

The 15 and 30 days of storage resulted in lower TOTOX values for cooked salami enriched with fruit powder (SI) compared with the scalded samples (SII), despite its higher initial lipid oxidation status. The efficacy of the fruit powder antioxidants (rich in phenolic compounds) is significantly influenced by the salami’s moisture content during cold storage. In the drier SI salami, the limited water content could concentrate the antioxidants within the lipid phase or at the lipid–water interface. This concentration, combined with restricted diffusion of pro-oxidants like oxygen, might allow the antioxidants to more effectively scavenge free radicals and chelate metal ions in their immediate vicinity, thereby sustaining their protective action for longer periods and limiting the extent of lipid oxidation. Conversely, in the wetter SII salami, the higher moisture content, while initially protective during heat treatment, might have allowed for greater mobility of oxygen and water-soluble pro-oxidants during cold storage. Although the fruit powders’ phenolic compounds still exert antioxidant activity (e.g., radical scavenging, metal chelation), their effectiveness might be diluted or overwhelmed by the faster rate of oxidative reactions facilitated by the higher moisture. This could lead to a quicker depletion of the antioxidants’ capacity, allowing lipid oxidation to advance more rapidly and resulting in higher TOTOX values over extended storage.

Regarding the inhibition potential against lipid oxidation during storage, BCP at a dose designed to provide a level of TPC of 300 mg GAE/kg of processed meat demonstrated the ability to effectively replace sodium nitrite in both SI and SII salami. Promising results for improving lipid oxidation resistance were also obtained by supplementing salami formulations with LP300. In SI-LP300 and SII-LP300, an inhibitory effect on oxidative degradation processes, slightly lower than that of sodium nitrite, was recorded at both 15 and 30 days of storage. This was reflected by TOTOX values of 2.84 (day 15) and 5.77 (day 30) for SI-LP300, compared with 2.56 (day 15) and 5.43 (day 30) for SI-CN. Similarly, SII-LP300 recorded TOTOX values of 4.17 (day 15) and 7.31 (day 30), compared with 4.05 (day 15) and 7.11 (day 30) for SII-CN.

Although there is currently no official TOTOX limit set by regulatory authorities for edible oils and fats, the voluntary GOED monograph [79] considers values below 26 to be acceptable. It has been suggested that TOTOX values below 10 can be considered indicative of superior lipid matrix quality [2].

By incorporating fruit powders, all SI formulations and most SII samples maintained TOTOX values below 10 even after 30 days of storage. The only exceptions were SII-SCP90 and SII-SCP-200, which recorded TOTOX values of 10.00 and 11.11, respectively. Our data demonstrated that replacing sodium nitrite with fruit powders in the salami recipe is a suitable application for improving oxidative quality both during processing and 30 days of cold storage. These findings align with other studies reporting a limitation in the increase in TOTOX values in stored meat products due to natural antioxidant addition, thereby demonstrating their potential to significantly slow down lipid oxidation [81].

#### 3.3.5. TBA Value

The TBA assay is a widely used method for evaluating the extent of lipid oxidation in food products. This test quantifies substances that react with thiobarbituric acid, typically aldehydes and ketones formed during the secondary phase of auto-oxidation when lipid peroxides break down [2]. Malondialdehyde (MDA), or 1,3-propanedial, is a key product of secondary oxidation of polyunsaturated fatty acids. In meat products, even small concentrations of MDA significantly contribute to rancid odors, making it a widely recognized indicator of lipid oxidation [2]. Currently, there’s no legally established limit for MDA concentrations in meat and meat products. However, different studies have proposed various thresholds for acceptability. Several authors suggest that malondialdehyde (MDA) levels between 2.0 and 2.5 mg/kg represent the upper threshold below which meat and meat products are generally not perceived as rancid [2,80]. Others consider levels above 1.0 mg/kg potentially unacceptable [82,83]. Campo et al. [84] proposed a limit of approximately 2.0 mg MDA/kg for the sensory acceptability of oxidized beef, while Hughes et al. [85] reported that meat products with TBA values between 2.60 and 3.11 mg MDA/kg were still deemed acceptable by consumers.

**Figure 5:**
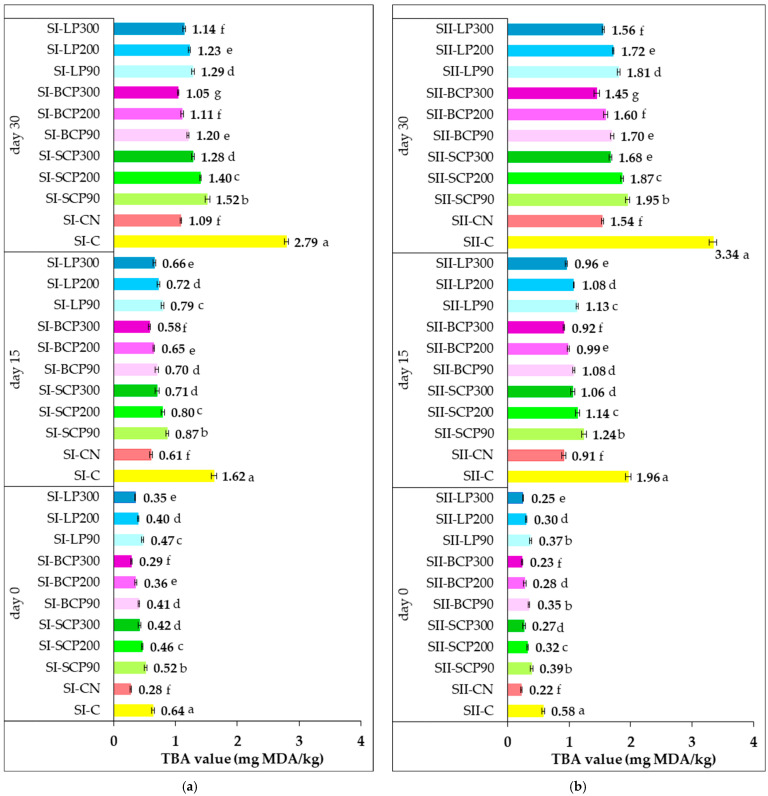
Impact of sodium nitrite and fruit powder supplementation on TBA value during cold storage of smoked and cooked salami (SI) (**a**) and smoked and scalded salami (SII) (**b**). SI-C, SII-C: Nitrite-free SI and SII salami; SI-CN, SII-CN: SI and SII salami with added sodium nitrite; SI-SCP90, SI-SCP200, SI-SCP300, SII-SCP90, SII-SCP200, SII-SCP300: Nitrite-free SI and SII salami with sour cherry powder added to provide a TPC of 90, 200 and 300 mg GAE/kg of meat; SI-BCP90, SI-BCP200, SI-BCP300, SII-BCP90, SII-BCP200, SII-BCP300: Nitrite-free SI and SII salami with blackcurrant powder added to provide a TPC of 90, 200 and 300 mg GAE/kg of meat; SI-LP90, SI-LP200, SI-LP300, SII-LP90, SII-LP200, SII-LP300: Nitrite-free SI and SII salami with lingonberry powder added to provide a TPC of 90, 200 and 300 mg GAE/kg of meat. Results are expressed as the mean ± standard deviation (SD) of three independent analyses. Means labeled with different letters within the same storage period are significantly different (one-way ANOVA, *p* < 0.05).

Figure 5 illustrates the effect of fruit powder incorporation into salami formulations on TBA values during cold storage (0, 15, and 30 days), in comparison with control samples without nitrite and those containing sodium nitrite.

TBA values revealed variations in lipid oxidation among salami formulas, attributable to thermal treatments during processing, fruit powder type and concentration, and cold storage duration. The initial TBA values in the supplemented salami were generally low. Specifically, SI-CN recorded 0.28 mg MDA/kg, and SII-CN recorded 0.22 mg MDA/kg. Values ranged from 0.29 to 0.52 mg MDA/kg in SI fruit-powder-enriched samples and from 0.23 to 0.39 mg MDA/kg in SII fruit-powder-enriched samples.

During the 15- and 30-day storage periods, the control samples (SI-C and SII-C) exhibited significantly higher TBA values compared with those containing sodium nitrite (SI-CN and SII-CN). The incorporation of fruit powder in different amounts also impacted the TBA values during storage (Figure 5).

After 30 days of storage, TBA values ranged from 1.05 to 1.52 mg MDA/kg for SI and 1.45 to 1.95 mg MDA/kg for SII. TBA values generally decreased with increasing fruit powder dose. At the end of the 30-day storage period, BCP-supplemented salami exhibited the lowest TBA values for both SI (1.05–1.20 mg MDA/kg) and SII (1.45–1.70 mg MDA/kg). These were followed by samples with LP (SI: 1.14–1.29 mg MDA/kg; SII: 1.56–1.81 mg MDA/kg), and then those enriched with SCP (SI: 1.28–1.52 mg MDA/kg; SII: 1.68–1.95 mg MDA/kg).

A closer look at the TBA values of positive control samples (SI-CN and SII-CN) and BCP300-enriched samples revealed no significant differences (*p* > 0.05) after processing (day 0) and after 15 days of cold storage. At the end of storage, no significant differences (*p* > 0.05) were observed in the TBA values of samples with nitrite and those supplemented with BCP200 and LP300. Incorporating BCP300 demonstrated a significantly greater inhibitory effect on secondary lipid oxidation compared with sodium nitrite added at 90 mg/kg of processed meat.

Across SI formulations, TBA values remained below 1 mg MDA/kg after 15 days of storage. Extending storage to 30 days increased TBA values, but they remained below 2 mg MDA/kg, suggesting these samples weren’t significantly affected by oxidation or rancidity. Even after 30 days, incorporating BCP200, BCP300, and LP300 into SI salami effectively inhibited secondary lipid oxidation, maintaining TBA values close to 1 mg MDA/kg. For SII samples, TBA values were higher than for SI samples, but they remained below the 2 mg MDA/kg threshold after 30 days of storage, even with fruit powders at doses providing a TPC of 90 mg GAE/kg in the processed meat. Incorporation of BCP300, BCP200, and LP300 into SII led to the lowest TBA values, ranging from 1.45 to 1.60 mg MDA/kg.

These results demonstrate the effectiveness of fruit powders in controlling the extent of lipid oxidation throughout the 30-day cold storage period recommended for the salami types investigated.

## 4. Conclusions

This study provides clear evidence that fruit-derived powders, particularly blackcurrant (BCP), lingonberry (LP), and sour cherry (SCP), can be used as effective natural antioxidants in salami production, offering a promising alternative to synthetic nitrites. Their efficacy is largely attributed to their rich phytochemical profiles, including phenolics, flavonoids, anthocyanins, and ascorbic acid, which significantly reduced lipid oxidation during both processing and cold storage. Among the powders tested, BCP, at a dose corresponding to 300 mg GAE/kg of meat, consistently provided the highest oxidative stability, comparable to or superior to that of sodium nitrite, as reflected in the lowest values of key oxidation markers (PV, pAV, TBA, and TOTOX), thus confirming its strong potential for use in clean-label formulations. LP and SCP also demonstrated notable antioxidant properties, suggesting their suitability for tailored applications based on specific product characteristics. The antioxidant effect was both dose-dependent and fruit powder-specific, with improved efficacy at higher phenolic levels. In addition, the processing method significantly influenced the oxidative performance: smoked and cooked salami (SI), with a lower moisture content, showed greater stability during storage compared to smoked and scalded salami (SII), where the higher moisture content facilitated more intense oxidative degradation over time. These findings highlight the importance of aligning antioxidant strategies not only with ingredient functionality but also with technological parameters. From an applied perspective, fortification with fruit powders, especially BCP, has been shown to be effective in maintaining oxidative stability below critical thresholds during 30 days of refrigerated storage, supporting their potential use in clean-label meat products. However, to fully validate the technological feasibility of replacing nitrites with fruit-derived antioxidants, further comprehensive studies are needed to assess the microbiological safety of these formulations. Future research is planned to evaluate the efficacy of these powders in controlling microbial growth during storage, particularly their potential to inhibit spoilage microorganisms and relevant foodborne pathogens in nitrite-free formulations. Such investigations will provide essential information on the microbiological safety and technological reliability of these natural additives, thereby supporting their effective integration into clean labeling strategies for industrial meat production.

## Figures and Tables

**Table 1:** Ingredients for SI salami manufacturing (control and fruit powder supplemented).

Sample	Pork Meat (g)	Pork Fat (g)	Salt (g)	Salt + 0.5% (*w*/*w*) Sodium Nitrite (g)	Spice Mixture (g)	SCP (g)	BCP (g)	LP (g)
SI-C	780	220	18	-	15	-	-	-
SI-CN	780	220	-	18	15	-	-	-
SI-SCP90	780	220	18	-	15	9.42	-	-
SI-SCP200	780	220	18	-	15	20.94	-	-
SI-SCP300	780	220	18	-	15	31.41	-	-
SI-BCP90	780	220	18	-	15	-	6.13	-
SI-BCP200	780	220	18	-	15	-	13.62	-
SI-BCP300	780	220	18	-	15	-	20.43	-
SI-LP90	780	220	18	-	15	-	-	7.83
SI-LP200	780	220	18	-	15	-	-	17.40
SI-LP300	780	220	18	-	15	-	-	26.10

**Table 2:** Ingredients for SII salami manufacturing (control and fruit powder supplemented).

Sample	Pork meat (g)	Pork Fat (g)	Salt (g)	Salt + 0.5% (*w*/*w*) Sodium Nitrite (g)	Spice Mixture (g)	SCP (g)	BCP (g)	LP (g)
SII-C	800	200	18	-	28	-	-	-
SII-CN	800	200	-	18	28	-	-	-
SII-SCP90	800	200	18	-	28	9.42	-	-
SII-SCP200	800	200	18	-	28	20.94	-	-
SII-SCP300	800	200	18	-	28	31.41	-	-
SII-BCP90	800	200	18	-	28	-	6.13	-
SII-BCP200	800	200	18	-	28	-	13.62	-
SII-BCP300	800	200	18	-	28	-	20.43	-
SII-LP90	800	200	18	-	28	-		7.83
SII-LP200	800	200	18	-	28	-	-	17.40
SII-LP300	800	200	18	-	28	-	-	26.10

**Table 3:** Phytochemical profile and antioxidant activity of fruit powder.

Fruit Powder	TPC (mg GAE/g d.w.)	TFC (mg QE/g d.w.)	TMA (mg C3G/100 g d.w.)	AsAc (mg/100 g d.w.)	FRAP (μM Fe^2+^/g d.w.)	DPPH (μM TE/g d.w.)
BCP	15.45 ± 0.51 ^a^	6.98 ± 0.19 ^a^	859.04 ± 1.89 ^a^	621.09 ± 1.73 ^a^	419.41 ± 1.39 ^a^	307.44 ± 1.57 ^a^
LP	12.07 ± 0.38 ^b^	6.11 ± 0.14 ^b^	312.58 ± 1.75 ^b^	64.52 ± 0.58 ^b^	370.47 ± 1.27 ^b^	278.65 ± 1.32 ^b^
SCP	10.01 ± 0.24 ^c^	4.85 ± 0.11 ^c^	509.73 ± 1.43 ^c^	45.91 ± 0.47 ^c^	294.59 ± 1.18 ^c^	220.71 ± 1.24 ^c^

BCP: blackcurrant powder; LP: lingonberry powder; SCP: sour cherry powder; TPC: total phenolic content, mg gallic acid equivalents (GAE)/g d.w.; TFC: total flavonoid content, mg quercetin equivalents (QE)/g d.w.; TMA: total monomeric anthocyanins, cyanidin-3-glucoside (C3G)/100 g d.w.; AsAc: ascorbic acid, mg/100 g d.w.; FRAP: ferric reducing antioxidant power, μM Fe^2+^/g d.w.; DPPH: 1,1-diphenyl-2-picrylhydrazyl, μM Trolox equivalents (TE)/g d.w. Results are expressed as the mean value of three independent analyses ± standard deviation (SD). Means in the same column bearing different superscripts are significantly different (one-way ANOVA, *p* < 0.05).

**Table 4:** Proximate composition and energy value of developed salami formulations (day 0).

Sample	Proximate Composition						Energy Value (kcal/100 g)
	Moisture (g/100 g)	Protein (g/100 g)	Lipids (g/100 g)	Ash (g/100 g)	NaCl (g/100 g)	CRB (g/100 g)	
SI-C	61.24 ± 0.06 ^a^	11.52 ± 0.06 ^a^	22.02 ± 0.06 ^a^	1.85 ± 0.02 ^e^	2.19 ± 0.03 ^a^	1.18	248.98
SI-CN	61.21 ± 0.04 ^a^	11.50 ± 0.05 ^a^	22.01 ± 0.07 ^a^	1.86 ± 0.01 ^e^	2.21 ± 0.02 ^a^	1.21	248.92
SI-SCP90	60.87 ± 0.03 ^d^	11.44 ± 0.05 ^a^	21.84 ± 0.04 ^b^	1.98 ± 0.01 ^d^	2.21 ± 0.01 ^a^	1.66	248.95
SI-SCP200	60.55 ± 0.04 ^e^	11.37 ± 0.03 ^a^	21.62 ± 0.06 ^c^	2.11 ± 0.02 ^b^	2.23 ± 0.02 ^a^	2.12	248.54
SI-SCP300	60.27 ± 0.05 ^g^	11.31 ± 0.05 ^a^	21.43 ± 0.07 ^c^	2.19 ± 0.03 ^a^	2.22 ± 0.01 ^a^	2.58	248.42
SI-BCP90	61.06 ± 0.05 ^b^	11.45 ± 0.04 ^a^	21.90 ± 0.05 ^b^	1.93 ± 0.02 ^d^	2.21 ± 0.03 ^a^	1.45	248.70
SI-BCP200	60.82 ± 0.04 ^d^	11.43 ± 0.05 ^a^	21.76 ± 0.06 ^b^	2.01 ± 0.01 ^c^	2.21 ± 0.01 ^a^	1.78	248.63
SI-BCP300	60.61 ± 0.05 ^e^	11.38 ± 0.03 ^a^	21.63 ± 0.05 ^c^	2.08 ± 0.02 ^b^	2.22 ± 0.03 ^a^	2.08	248.50
SI-LP90	60.95 ± 0.03 ^c^	11.44 ± 0.04 ^a^	21.87 ± 0.06 ^b^	1.90 ± 0.02 ^d^	2.22 ± 0.01 ^a^	1.61	249.08
SI-LP200	60.69 ± 0.04 ^e^	11.40 ± 0.05 ^a^	21.69 ± 0.06 ^c^	1.97 ± 0.03 ^d^	2.23 ± 0.02 ^a^	2.01	248.91
SI-LP300	60.46 ± 0.03 ^f^	11.37 ± 0.04 ^a^	21.53 ± 0.05 ^c^	2.03 ± 0.02 ^c^	2.24 ± 0.03 ^a^	2.36	248.75
SII-C	63.27 ± 0.05 ^a^	12.63 ± 0.04 ^a^	19.51 ± 0.03 ^a^	1.92 ± 0.03 ^d^	2.10 ± 0.02 ^a^	0.57	228.39
SII-CN	63.28 ± 0.07 ^a^	12.65 ± 0.06 ^a^	19.53 ± 0.04 ^a^	1.93 ± 0.02 ^d^	2.13 ± 0.03 ^a^	0.48	228.27
SII-SCP90	62.88 ± 0.03 ^d^	12.57 ± 0.05 ^a^	19.37 ± 0.05 ^b^	2.07 ± 0.02 ^b^	2.12 ± 0.01 ^a^	0.99	228.59
SII-SCP200	62.56 ± 0.04 ^f^	12.50 ± 0.04 ^a^	19.21 ± 0.06 ^b^	2.18 ± 0.03 ^b^	2.13 ± 0.02 ^a^	1.42	228.59
SII-SCP300	62.31 ± 0.05 ^g^	12.43 ± 0.05 ^a^	19.02 ± 0.05 ^b^	2.28 ± 0.04 ^a^	2.15 ± 0.03 ^a^	1.81	228.13
SII-BCP90	63.05 ± 0.04 ^b^	12.59 ± 0.03 ^a^	19.43 ± 0.03 ^b^	2.02 ± 0.02 ^c^	2.10 ± 0.03 ^a^	0.81	228.45
SII-BCP200	62.82 ± 0.05 ^d^	12.55 ± 0.06 ^a^	19.30 ± 0.04 ^b^	2.11 ± 0.02 ^b^	2.11 ± 0.02 ^a^	1.11	228.35
SII-BCP300	62.61 ± 0.04 ^f^	12.51 ± 0.05 ^a^	19.19 ± 0.03 ^b^	2.19 ± 0.01 ^b^	2.12 ± 0.01 ^a^	1.38	228.28
SII-LP90	62.95 ± 0.03 ^c^	12.59 ± 0.03 ^a^	19.40 ± 0.05 ^b^	1.99 ± 0.02 ^c^	2.10 ± 0.03 ^a^	0.97	228.85
SII-LP200	62.71 ± 0.04 ^e^	12.54 ± 0.05 ^a^	19.25 ± 0.07 ^b^	2.08 ± 0.02 ^b^	2.12 ± 0.01 ^a^	1.31	228.60
SII-LP300	62.48 ± 0.05 ^f^	12.48 ± 0.04 ^a^	19.11 ± 0.06 ^b^	2.15 ± 0.03 ^b^	2.13 ± 0.02 ^a^	1.65	228.52

CRB: Carbohydrates. SI-C, SII-C: Nitrite-free SI and SII salami; SI-CN, SII-CN: SI and SII salami with added sodium nitrite; SI-SCP90, SI-SCP200, SI-SCP300, SII-SCP90, SII-SCP200, SII-SCP300: Nitrite-free SI and SII salami with sour cherry powder added to provide a TPC of 90, 200 and 300 mg GAE/kg of meat; SI-BCP90, SI-BCP200, SI-BCP300, SII-BCP90, SII-BCP200, SII-BCP300: Nitrite-free SI and SII salami with blackcurrant powder added to provide a TPC of 90, 200 and 300 mg GAE/kg of meat; SI-LP90, SI-LP200, SI-LP300, SII-LP90, SII-LP200, SII-LP300: Nitrite-free SI and SII salami with lingonberry powder added to provide a TPC of 90, 200 and 300 mg GAE/kg of meat. Results are expressed as the mean ± standard deviation (SD) of three independent analyses. Means in the same column bearing different superscripts are significantly different (one-way ANOVA, *p* < 0.05).

**Table 5:** Changes in the inhibition of oxidation (IO) during cold storage of smoked and cooked salami (SI) and smoked and scalded salami (SII), supplemented with sodium nitrite and fruit powder.

Sample	IO (%)	
	Day 15	Day 30
SI-CN	72.72 ± 0.24 ^a^	53.59 ± 0.23 ^a^
SI-SCP90	47.75 ± 0.21 ^i^	24.68 ± 0.11 ^i^
SI-SCP200	58.83 ± 0.16 ^g^	34.85 ± 0.15 ^h^
SI-SCP300	66.85 ± 0.19 ^d^	42.93± 0.18 ^e^
SI-BCP90	55.73 ± 0.12 ^h^	41.90 ± 0.20 ^f^
SI-BCP200	63.16 ± 0.20 ^f^	47.72 ± 0.24 ^c^
SI-BCP300	71.10 ± 0.22 ^b^	53.82 ± 0.26 ^a^
SI-LP90	55.77 ± 0.17 ^h^	36.33 ± 0.16 ^g^
SI-LP200	65.28 ± 0.19 ^e^	44.16 ± 0.19 ^d^
SI-LP300	70.45 ± 0.26 ^c^	52.22 ± 0.21 ^b^
SII-CN	54.83 ± 0.16 ^c^	42.43 ± 0.15 ^b^
SII-SCP90	36.35 ± 0.23 ^i^	18.22 ± 0.10 ^i^
SII-SCP200	48.20 ± 0.27 ^f^	25.46 ± 0.12 ^h^
SII-SCP300	53.42 ± 0.19 ^d^	33.09 ± 0.16 ^f^
SII-BCP90	42.71 ± 0.15 ^g^	37.08 ± 0.20 ^d^
SII-BCP200	47.82 ± 0.25 f	39.58 ± 0.22 ^c^
SII-BCP300	57.08 ± 0.26 ^a^	43.66 ± 0.26 ^a^
SII-LP90	39.99 ± 0.17 ^h^	29.21 ± 0.19 ^g^
SII-LP200	49.04 ± 0.20 ^e^	35.66 ± 0.21 ^e^
SII-LP300	55.70 ± 0.22 ^b^	42.38 ± 0.14 ^b^

SI-C, SII-C: Nitrite-free SI and SII salami; SI-CN, SII-CN: SI and SII salami with added sodium nitrite; SI-SCP90, SI-SCP200, SI-SCP300, SII-SCP90, SII-SCP200, SII-SCP300: Nitrite-free SI and SII salami with sour cherry powder added to provide a TPC of 90, 200 and 300 mg GAE/kg of meat; SI-BCP90, SI-BCP200, SI-BCP300, SII-BCP90, SII-BCP200, SII-BCP300: Nitrite-free SI and SII salami with blackcurrant powder added to provide a TPC of 90, 200 and 300 mg GAE/kg of meat; SI-LP90, SI-LP200, SI-LP300, SII-LP90, SII-LP200, SII-LP300: Nitrite-free SI and SII salami with lingonberry powder added to provide a TPC of 90, 200 and 300 mg GAE/kg of meat. Results are expressed as the mean ± standard deviation (SD) of three independent analyses. Means labeled with different letters within the same storage period are significantly different (one-way ANOVA, *p* < 0.05).

## Data Availability

The original data presented in the study are openly available at the University of Life Sciences “King Mihai I” from Timisoara.
